# Activated carbons with extremely high surface area produced from cones, bark and wood using the same procedure†

**DOI:** 10.1039/d3ra00820g

**Published:** 2023-05-12

**Authors:** Gui Li, Artem Iakunkov, Nicolas Boulanger, Oana Andreea Lazar, Marius Enachescu, Alejandro Grimm, Alexandr V. Talyzin

**Affiliations:** a Department of Physics, Umeå University Umeå SE-90187 Sweden alexandr.talyzin@umu.se; b Center for Surface Science and Nanotechnology, University Politehnica of Bucharest Splaiul Independentei 313 Bucharest 060032 Romania; c Department of Forest Biomaterials and Technology, Biomass Technology Centre, Swedish University of Agricultural Sciences Umeå SE-901 83 Sweden

## Abstract

Activated carbons have been previously produced from a huge variety of biomaterials often reporting advantages of using certain precursors. Here we used pine cones, spruce cones, larch cones and a pine bark/wood chip mixture to produce activated carbons in order to verify the influence of the precursor on properties of the final materials. The biochars were converted into activated carbons with extremely high BET surface area up to ∼3500 m^2^ g^−1^ (among the highest reported) using identical carbonization and KOH activation procedures. The activated carbons produced from all precursors demonstrated similar specific surface area (SSA), pore size distribution and performance to electrodes in supercapacitors. Activated carbons produced from wood waste appeared to be also very similar to “activated graphene” prepared by the same KOH procedure. Hydrogen sorption of AC follows expected uptake *vs.* SSA trends and energy storage parameters of supercapacitor electrodes prepared from AC are very similar for all tested precursors. It can be concluded that the type of precursor (biomaterial or reduced graphene oxide) has smaller importance for producing high surface area activated carbons compared to details of carbonization and activation. Nearly all kinds of wood waste provided by the forest industry can possibly be converted into high quality AC suitable for preparation of electrode materials.

## Abstract 2

Identical carbonization and KOH activation procedures result in very similar activated carbons with ultra-high BET surface area (up to ∼3500 m^2^ g^−1^) when applied to several types of precursors: pine, spruce, and larch cones, and bark/wood chips.

## Graphical abstract

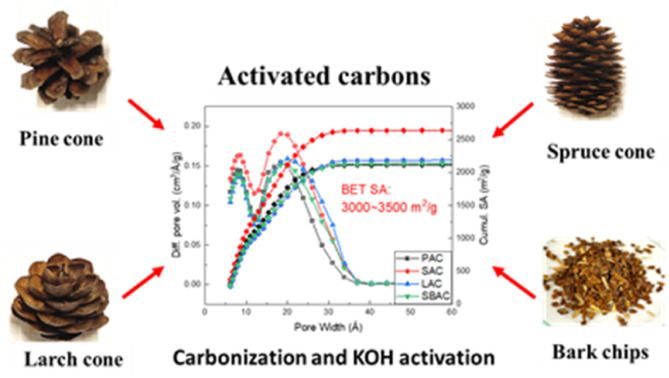

## Introduction

Activated carbons (AC) are materials produced on an industrial scale and used in a variety of applications, *e.g.* in water purification^1^ and as a main component of electrode materials in industrial supercapacitors.^2^ High specific surface area (SSA) is one of the main parameters required in ideal supercapacitor (SC) electrode materials. Several other parameters (*e.g.* high electrical conductivity, pore size, methods of compaction *etc.*) are important for better performance of electrodes but high surface area is the key element of modern supercapacitor materials.^3–6^ AC and its composites with other nanostructured carbons were very actively tested in water treatment and energy storage applications in a recent decade.^7,8^

Extremely high surface area is also required in sorption applications, *e.g.* for improved hydrogen storage and for efficient removal of various contaminants from aqueous waste solutions. It is well known by now that physisorption of hydrogen is almost linearly proportional to SSA both at ambient and liquid nitrogen temperature.^9^ This trend is valid for all kinds of nanostructured carbon materials including AC, carbon nanotubes (CNT) and graphene-related materials.^10,11^

Many nanostructured carbon materials had emerged over past two decades, but ACs remain to be materials with highest SSA values, in some reports as high as ∼3000–3900 m^2^ g^−1^. It should be noted that at least part of reports for extremely high SSA of AC (>3500 m^2^ g^−1^) is likely related to incorrect procedures applied to analysis of sorption isotherms. Verified procedure specific for microporous materials has been designed by J. Rouquerol *et al.*^12^ The procedure allows reading reproducible values of SSA in microporous materials by selecting specific interval of relative pressures for a BET plot.^12^ However, there is no doubt that AC with BET SSA exceeding 3000 m^2^ g^−1^ is possible to synthesize using carefully tuned activation procedures.^5,13^

Activated carbons are commonly produced using large variety of bio precursors, most commonly plants, wood or different kinds of organic waste, *e.g.* old tires,^14^ food waste,^15^ spent tea leaves,^16^ lignin,^17^ olive pits,^18,19^ waste tea^20^ and coffee,^19^ corn cob,^21^ coconut shell,^13^ sawdust.^22^ In this case converting bio precursors into AC is not only providing useful materials but also contributes to waste recycling.

High surface area is achieved after carbonization of bio precursor by annealing under inert gas and activation of so prepared biochar using variety of methods. Most common in industrial applications is steam activation typically providing AC with surface area of about 2000 m^2^ g^−1^.^23–25^ The highest BET SSA is found in AC produced using KOH activation,^26^ and the method is based on mixing biochar with KOH in different proportions and annealing the mixture at elevated temperatures (∼700–1000 °C). Possibly the most studied high surface area AC (∼3000 m^2^ g^−1^) was commercially produced for period of time using petroleum coke under the name “Maxsorb”.^27^ AC with similarly high surface area was produced also starting from several bio precursors and, more recently, using reduced graphene oxide (under the name “Activated Graphene”).^28–35^

It should be noted that achieving BET surface area values on the level of 3000 m^2^ g^−1^ requires careful tuning of several parameters of KOH activation procedure. Most important parameters are: activation temperature, KOH to carbon ratio and homogeneous distribution of KOH in the carbon precursor.^3,10,11^

A very high surface area has been reported recently for AC produced using KOH activation of pinecone biochars.^36–40^ Pinecone derived AC have been studied previously for removal of contaminants from aqueous solutions by adsorption.^22,36,38,40–43^ and for the preparation of supercapacitors electrodes.^39,44–48^ However, most of these studies were performed with AC with SSA (1000–2000 m^2^ g^−1^)^39,45,48^ and single report on material with ∼2500 m^2^ g^−1^.^44^

Recently we demonstrated that KOH activation tuned previously for preparation of “activated graphene” could be used to produce AC with similarly high BET SSA of about ∼3000 m^2^ g^−1^ starting from pinecone biochar.^49^ The pinecone AC showed similar to “activated graphene” pore size distribution and good performance in supercapacitor electrodes produced by both pellet forming and spray deposition^49^ using aqueous dispersions.^50,51^

The pinecone derived AC provides an example of inexpensive alternative for sorption applications and electrode preparation for energy storage applications. However, it is not clear how much the properties of the pinecone AC are determined by the precursor biomaterial and by details of KOH procedure. Literally hundreds of studies have been published in recent years claiming advantages of this or other bio precursors for preparation of AC. However, systematic studies focused on using identical activation methods with biochars of different origins are very scarce and not available (to the best of our knowledge) for high surface area AC (∼3000 m^2^ g^−1^).

In this study, we produced AC with an exceptionally high BET surface area up to ∼3500 m^2^ g^−1^ using several common in Scandinavia by-products of wood industry, typically considered as waste. Identical KOH activation was applied to biochars produced using identical procedure starting from pinecones, spruce cones, larch cones and bark/wood chips. The ACs produced from all tested precursors appeared to be very similar except for somewhat different ash content. Therefore, it is demonstrated that details of KOH activation are a lot more important for synthesis of high surface area AC compared to the kind of bio precursor. The AC was used as electrode material in supercapacitors demonstrating excellent energy storage parameters. Hydrogen sorption was measured for the high surface area AC using volumetric and gravimetric tank methods. It is demonstrated that adding AC to the storage tank provides ∼9% increase in amount of stored hydrogen compared to compressed H_2_.

## Experimental details

### Materials preparation

Four types of cones from coniferous trees were picked up in the Umeå (Sweden) region. Some tests were performed with full cones but more typically, only the cone scales were separated for further carbonization and activation. The cones or scales were cleaned with ethanol and distilled water repeatedly until the water was clear in order to get rid of contaminants. After drying in the oven for one night, the samples were carbonized in the tube furnace at 500 °C for 2 hours (heating rate 5 °C min^−1^, cooling down naturally) under argon flow. The biochars produced by carbonization were ground into small particles and then mixed with KOH (mass ratio = 8 : 1) in 75% ethanol (200 ml). The mixture was dried in vacuum oven after stirring overnight to provide homogeneous mixing of KOH with the biochar particles.

The two step KOH procedure used in this study for producing AC with extremely high surface area was earlier optimized using experiments with rGO precursor.^3,10,11^ It should be noted that precise control of KOH to carbon ratio is required for achieving highest surface area. This control is impossible in one step procedure where KOH is added directly to bioprecursor. The one step procedure results in materials with significantly smaller surface area (<2000 m^2^ g^−1^).^49,52^ Using ethanol as solvent for KOH is justified by rapid evaporation and more homogeneous distribution of KOH in the powder biochar sample.

The activation was performed using following temperature profile: heating up under argon flow from room temperature to 200 °C and maintaining at this temperature for 30 min in order to remove water and volatile impurities; slow heating up to 850 °C (2 hours and 40 minutes); maintaining at 850 °C for 3 hours before cooling down slowly. The activated materials were rinsed with acetic acid solution (10 vol%) in order to remove acid soluble impurities. The last step was vacuum filtration of the solution with 4 L distilled water to wash out remaining KOH and water soluble impurities.

Typically the starting amount of precursor was about 15–16 g resulting after carbonization in about 9–11 g of biochar. Four grams of biochar were typically used in each activation batch resulting in about 1–1.5 g of AC material. The overall yield of two step procedure is therefore, about 7–12%.

The four kinds of activated carbon from the cones of pine (Scots, Pinus sylvestris), spruce (Picea abies), silver spruce (Picea pungens) and larch (Larix decidua) trees were named as PAC, SAC, SSAC and LAC, respectively. Another type of tested precursor was bark with wood chips produced as waste in a wood processing company. The bark chips from Scots pine (Pinus sylvestris) provided by Ekenäs Timber AB, Ekenässjön, Sweden, were ground and sieved before carbonization. The samples with a particle size of 2–3 mm were used for producing the activated carbon, named as SBAC.

### Characterization

N_2_ adsorption/desorption isotherms were recorded at liquid nitrogen temperature and analysed to calculate surface area and pore size distribution of materials. All the samples were degassed at 150 °C for 22 hours prior to recording the isotherms. Slit-pore QSDFT model was used to calculate cumulative surface area, the pore volume and size distribution of pores. SEM (Zeiss Merlin FEG-SEM microscope) and TEM (Hitachi HD 2700 Scanning Transmission Electron Microscope) were used for surface imaging of the carbonized bio-char and activated carbons. Thermogravimetric analysis (TGA) was carried out using a Mettler Toledo TGA/DSC1 STARe System. The weight change scans were collected for temperature range from RT to 700 °C at 5 K min^−1^ under air and N_2_ flow (40 ml min^−1^).

XPS spectra were recorded using a Kratos Axis Ultra electron spectrometer with a monochromatic Al Kα source at 150 W and processed by the Kratos software. Raman spectra were obtained from Raman spectrometer (Ranishaw Qontor) equipped with 532 nm solid state laser.

### Preparation of supercapacitor electrodes and electrochemical characterization

Supercapacitor devices were prepared and tested using two types of electrodes: first method to prepare electrodes was standard palletization of AC with binder and second method was to use aqueous dispersions.

#### Pellet preparation

AC powder was mixed with carbon black and binder with mass ratio of 8 : 1 : 1. Then the mixture was pressed into pellets at around 80 MPa with an area of 1.2 cm^2^ and a weight around 7 mg. The pellets were additionally annealed at 200 °C for 1 hour in air before performing the electrochemical measurement.

#### Dispersion preparation

AC powder was mixed with fumed silica, carbon black and graphene oxide powder with a mass ratio at 10 : 1 : 1 : 1 following earlier described procedure.^50,51^ The activated material was ball milled for 5 minutes and then the other three components were added with 5 ml distilled water and another 5 minutes ball milling applied. Another 5 ml water was to wash the container after ball milling. The concentration of the final dispersion was set to 20 mg ml^−1^. The dispersion was blade coated on stainless steel and aluminum foil for making electrodes. The electrodes were annealed as mentioned before in order to convert non-conductive graphene oxide into electrically conductive reduced graphene oxide (rGO).

Cyclic voltammetry (CV), Galvanostatic charge–discharge cycling (GD), electrochemical impedance spectroscopy (EIS) and cycling stability tests were performed using Iviumstat potentiostat and symmetric cells with two identical AC-based electrodes, two stainless steel current collectors and a glass fiber membrane separator (Whatsman). 6 M KOH solution and 1 M tetraethylammonium tetrafluoroborate in acetonitrile (TEA TEF) electrolytes were used. For EIS, 10 mV sinusoidal amplitude alternating voltage at frequencies from 1 MHz to 10 MHz was applied. The stability test with 10 000 charging and discharging cycles was done at 3 A g^−1^ in aqueous electrolyte and at 5 A g^−1^ in organic electrolyte.

### Hydrogen sorption measurements

H_2_ uptake (excess wt%) was estimated using two methods: volumetric^11,53^ and “gravimetric tank method” developed in our earlier study.^54^ Volumetric tests were performed using Hiden Isochema Intelligent Manometric Instrument at ambient temperature and temperature of liquid nitrogen 77 K. The samples were degassed for 16 h before analysis at 323 K and high vacuum conditions (0.4 mbar). Hydrogen adsorption isotherms were recorded up to 120 bar of H_2_ at ambient pressure and 90 bar at 77 K.

The main purpose of gravimetric tank method (GTM) is to evaluate directly how much hydrogen is stored in the given tank with and without hydrogen storage material. The method involves a direct weight measurement of small volume tank. The weight of the tank is measured first in empty state with vacuum inside, next for the same tank filled with hydrogen under required pressure (120 bar) and finally the tank filled with material after dozing with hydrogen at 120 bar. The weight of hydrogen inside a material-filled test tank is compared then to the weight of the same tank filled with compressed hydrogen in the absence of loaded material.

The hydrogen storage capacity of material is then characterized relative to the amount of hydrogen stored in pure compressed gas using parameter introduced in our earlier study under the name hydrogen storage “Gain”. For example, the tank filled with material and Gain value of 10% will provide a 10% increase in the weight of hydrogen compared to material-free tank filled with hydrogen gas the same *P*–*T* conditions.

The test tank is sealed using standard valve and disconnected from the hydrogen dozing system for the weight measurements.^54^ Weight measurements were performed using a Radwag AS 510.3Y balance with a maximum load of 510 g and readability of 0.1 mg. All weight measurements were repeated 10 times and averaged to improve repeatability (see ESI† for details).

The GTM requires relatively large amount of material for reliable evaluation of H_2_ storage capacity of material (limited by the balance repeatability). The AC sample used in the H_2_ sorption test was produced in a separate synthesis batch using larch cones as a precursor. The weight of the sample after degassing was 0.9136 ± 0.0004 g. The inner volume of this assembly measured by pycnometry was 10.49 ml with a useful volume (volume occupied by material) of ∼7.2 ml, sufficient to accommodate about 1 g of AC material. Gas dosing was performed using a Hiden Isochema IMI volumetric system with a typical pressure of 120 bar and temperature of 23 °C. The sample was covered by a layer of silica wool in order to prevent outflow of material in the process of gas compression and pressure release.

Total Storage Density (TDS) specific for the given tank is defined as

1 TSD_tank_ = *m*_total_/*V*_tank_

where *m*_total_ – the total weight of hydrogen in a tank, *V*_tank_ – total volume of tank.

The material related Tank Storage Density (TSD_mat_) is defined as a density of H_2_ gas within the volume occupied by the sample (useful volume *V*_useful_). The dead volume (*V*_dead_) is the part of the tank not filled with material due to sample size limitations (incompletely filled volume) and includes the volume of connection pipes and the volume inside of the valve. The total weight of hydrogen in the material filled volume (TSD_mat_) can be calculated by subtracting the amount of hydrogen stored as gas in the dead volume *V*_dead_ from the total storage *m*_total_(1):

2 TSD = (*m*_total_ − *V*_dead_ × *ρ*_H_2__(*P*,*T*))/*V*_useful_

Here *m*_total_ – the total weight of hydrogen in a tank, *V*_dead_ – a volume of tank which is not filled with material and occupied by free hydrogen, *ρ*_H_2__(*P*,*T*) – density of hydrogen, *V*_useful_ – useful volume of tank, which is occupied by material (calculated using its bulk density). More details about calculation of *m*_total_, *V*_useful_ and *V*_dead_ are provided in earlier publication.^53^

Gain value shows the change in the density of hydrogen inside of the test tank due to addition of material relative to the density of hydrogen gas at given *P*–*T* conditions. It is expressed in percent. A positive value of Gain will be found for materials, which store hydrogen better compared to compressed gas and negative for materials, which store hydrogen less well. This parameter is calculated as the difference in hydrogen storage between a tank filled with a material and an empty tank with the same volume (3).

3 Gain = 100% × (TSD − *ρ*_H_2__(*P*,*T*))/*ρ*_H_2__(*P*,*T*)

The Gain (*G*_tank_) calculated using the total volume of the tank (TSD_tank_) in eqn (3), is specific only for a certain construction of the test tank and dead volume. The material-related Gain (*G*_mat_) can be calculated using eqn (3) with TSD_mat_, the known bulk density of the material and the mass of material (see ESI† for details). The value of Gain discussed below is calculated as *G*_mat_. More details of GTM details (*e.g.* error analysis) and related calculations can be found in our earlier publication.^53^

## Results and discussion

### Characterization of AC produced from several precursors

**Fig. 1:**
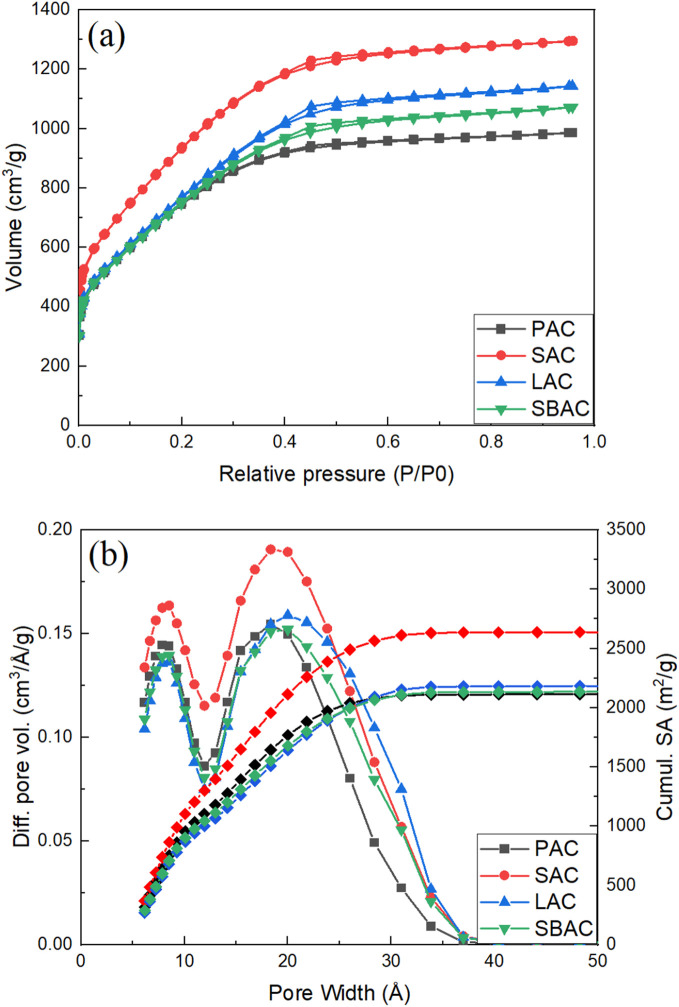
(a) N_2_-sorption isotherms; (b) pore size distribution (left axis) and cumulative surface area (right axis) of PAC, SAC, SBAC and LAC samples (see Table 1 for meaning of abbreviations).

Identical thermal carbonization and KOH activation procedures were applied in this study to bio precursors highly abundant and typically considered as waste in a forest industry: pinecones, spruce cones, larch cones and bark/wood chip mixture (Table 1). High surface area AC was produced using all these precursors where the highest BET SSA value of 3565 m^2^ g^−1^ and pore volume (1.88 cm^3^ g^−1^) for material prepared was from spruce scales. The Spruce Activated Carbon (SAC) appeared also to be one of the purest consisting of 96.82% carbon as proved by TGA test performed in air. Remarkably, pore size distribution of all AC's produced from forest waste in our experiments appeared to be very similar exhibiting rather narrow peak due to micropores (∼8 Å width) and a bit broader peak due to mesopores (∼20–25 Å width) (Fig. 1).

**Table 1:** Properties of AC samples synthesized using different bio precursors. Columns from the left to right: name of sample and bio precursor; precursor image; overall mass yield of AC; BET SSA of biochars (bch); BET SSA of activated samples, BET SSA calculated for carbon part of sample (excluding ash); total pore volume and micropore volume (the value in brackets) calculated using QSDFT model, carbon content according to TGA data

Samples	Image	Yield (∼%)	SSA bch m^2^ g^−1^	SSA AC m^2^ g^−1^	SSA m^2^ g^−1^ ash-free	Pore volume cm^3^ g^−1^	Carbon content %
PAC (pine cone)	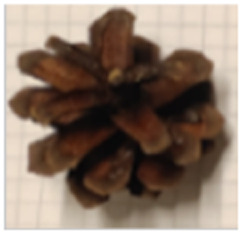	10.5	212	2813	3125	1.43 (0.98)	90.03
SAC (spruce cone)	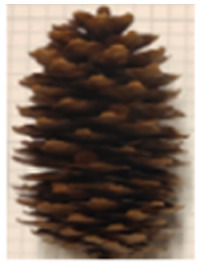	12.0	92	3565	3682	1.88 (1.19)	96.82
LAC (larch cone)	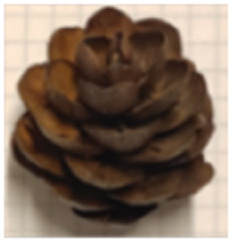	11.9	150	2981	3498	1.67 (0.93)	85.22
SSAC (silver spruce cone)	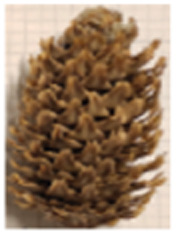	11.2	90	3497	3617	2.01 (1.05)	96.68
SBAC (pine bark chips)	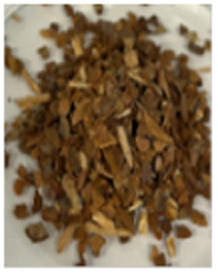	12.4	171	2856	2887	1.56 (0.94)	98.91
Full spruce cone	—	10.8	—	3067	3243	1.59 (1.05)	94.57

Rather similar pore size distribution observed in all samples independently on the type of precursor points out to essentially the same nature of AC. Moreover, the pore size distribution and BET SSA of AC prepared in our experiments are rather similar to those of “activated graphene” (Fig. S3†), material prepared by KOH activation of rGO and characterized in detail in earlier studies.^10,29,30^

**Fig. 2:**
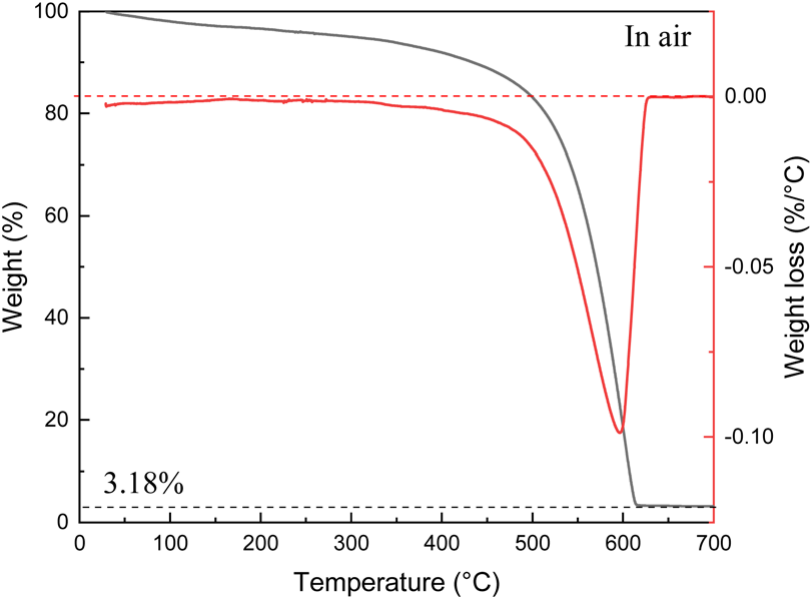
TGA data recorded for the thermal decomposition of SAC sample in air: weight loss curve (black) and differential curve (red). The value in % refers to amount of non-combustible impurity (ash content).

The scatter in the SSA values measured from samples listed in Table 1 is found to correlate with amount of non-combustible impurities (ash) determined using TGA traces recorded under air up to 700 °C (example shown in Fig. 2).

**Fig. 3:**
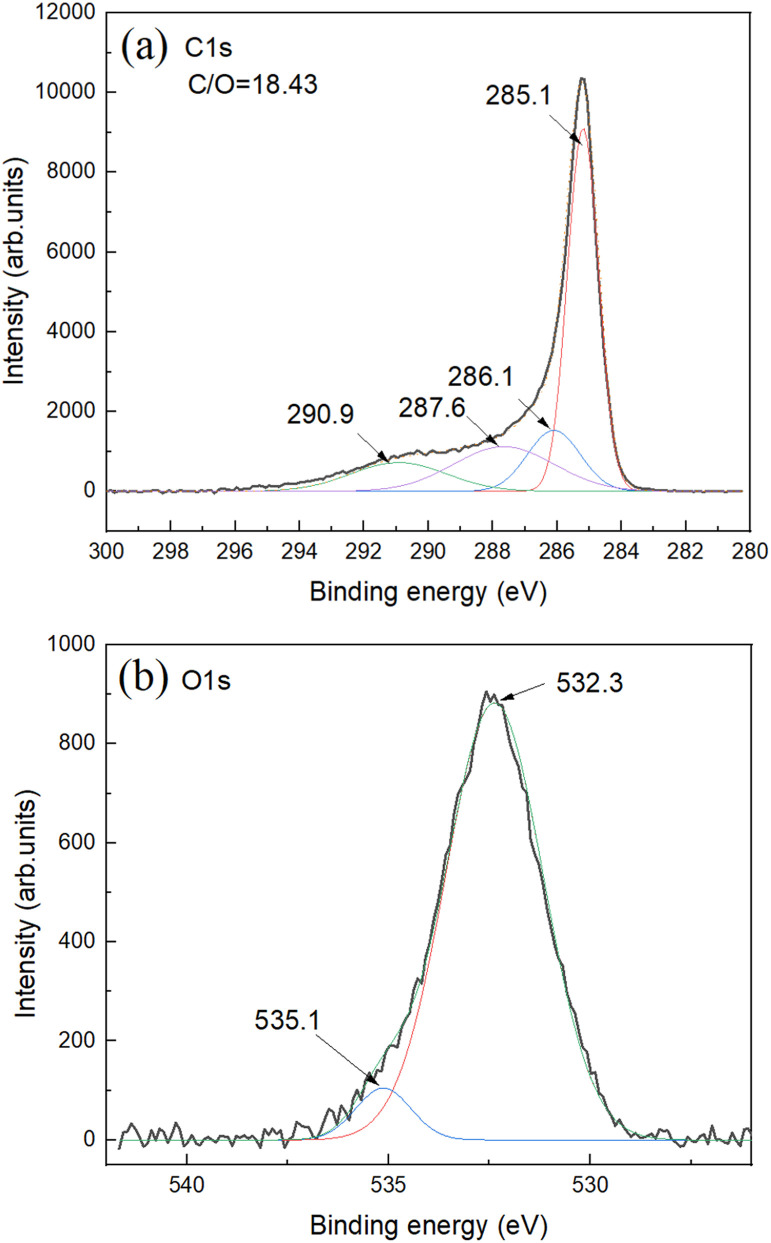
XPS spectra for SAC sample: (a) C 1s and (b) O 1s. Survey XPS spectra can be found in ESI File.† The overall C/O ratio is calculated using integrals from C 1s and O 1s parts.

The C 1s XPS spectra show main peak from carbon in non-oxidized state (285.1 eV) and small components typically assigned to carbon with oxygen functional groups (Fig. 3a).

Oxygen part of spectra shows one major feature assigned to carbon–oxygen bonds and small feature due to oxygen bound to aluminium impurity (Fig. 3b). Aluminium impurity is likely from the ceramic of boats and tube furnace. Survey XPS spectra do not reveal other impurities (Fig. S8†). The Al impurity was found to be in a form of Al (OH)_3_ as revealed by XRD. The AC itself showed no diffraction peaks in XRD patterns with some diffuse scattering in the low angle region (ESI File†).

Note that XPS is surface method and needs to be used for evaluation of bulk impurity content with some caution. Since the starting cones were collected outdoors in natural environments, these bio precursors are always contaminated with impurity particles, some non-combustible inorganic contaminants are always present also in the wood itself and finally some contamination is added in a process of carbonization/activation (*e.g.* materials from ceramic boats and furnace tube). Therefore, it is informative to calculate SSA value for only carbon part of samples excluding the weight of inorganic non-combustible contaminants. The SSA values in the range ∼2800–3500 cm^3^ g^−1^ were found for samples listed in the Table 1. The AC produced from bark shows smallest SSA value. The difference in SSA AC produced using cones is at least partly related to the ash content. Excluding non-combustible part from calculation provides “ideal” SSA values of cone derived AC in the range ∼3100–3700 m^2^ g^−1^. Some similarity in pore size distribution and surface area could possibly be anticipated for AC produced starting from cones of different kinds of tree species. However, nearly identical properties were found also for AC produced using bark/wood chip mixture, the precursor very different compared to cones and collected directly at the waste disposal locality.

**Fig. 4:**
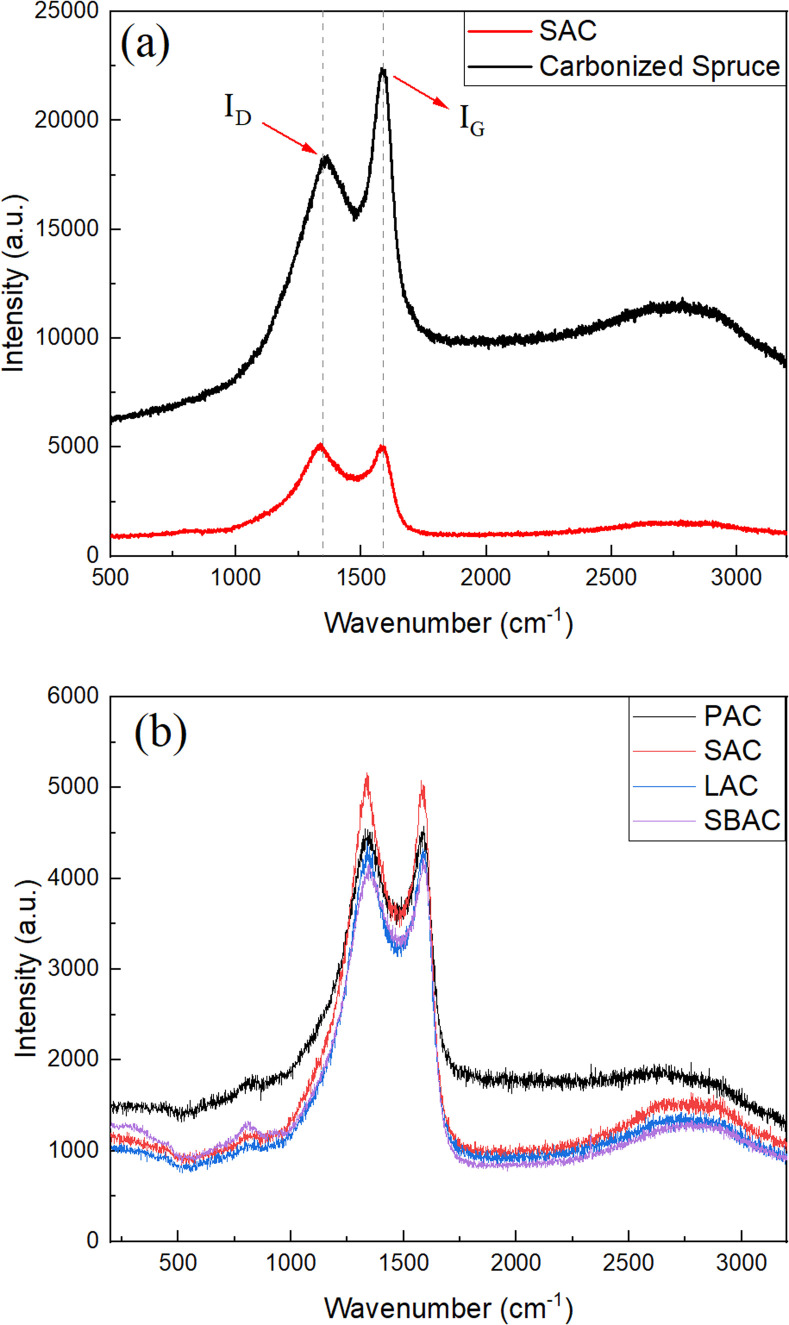
Raman spectra recorded from (a) biochar (carbonized spruce cones) and SAC sample. Peaks corresponding to G- and D-modes are indicated; (b) Raman spectra recorded from SAC, LAC, SBAC and PAC samples.

Characterization of AC produced using precursors listed in the Table 1 was performed also using Raman spectroscopy (Fig. 4) and SEM/TEM imaging. The Raman spectra of biochars and AC's are typical for amorphous carbon materials exhibiting broad D- and G-modes. Raman spectra of SAC and spruce biochar are shown in the Fig. 4a for the region from 500 cm^−1^ to 3200 cm^−1^. The value of intensity ratio *I*_D_/*I*_G_ is changed from 3.04 in the biochar to 4.75–5.78 as a result of KOH activation (Table 2), indicating higher purity of carbon in the activated materials. Once again, Raman spectra recorded from all types of AC showed similarity with relative intensity *I*_D_/*I*_G_ nearly identical independently on the used precursor.

**Table 2:** Raman peak position for D- and G-modes, areas of the peaks, D-mode to G-mode intensity ratios of PAC, SAC, LAC, SBAC and carbonized spruce

Samples	D peak (cm^−1^)	G peak (cm^−1^)	*I*_D_/*I*_G_
PAC	1336.1	1591.5	5.78
SAC	1334.0	1589.3	4.75
LAC	1334.9	1590.4	4.83
SBAC	1334.5	1590.2	4.90
Carbonized spruce	1356.3	1592.0	3.04

**Fig. 5:**
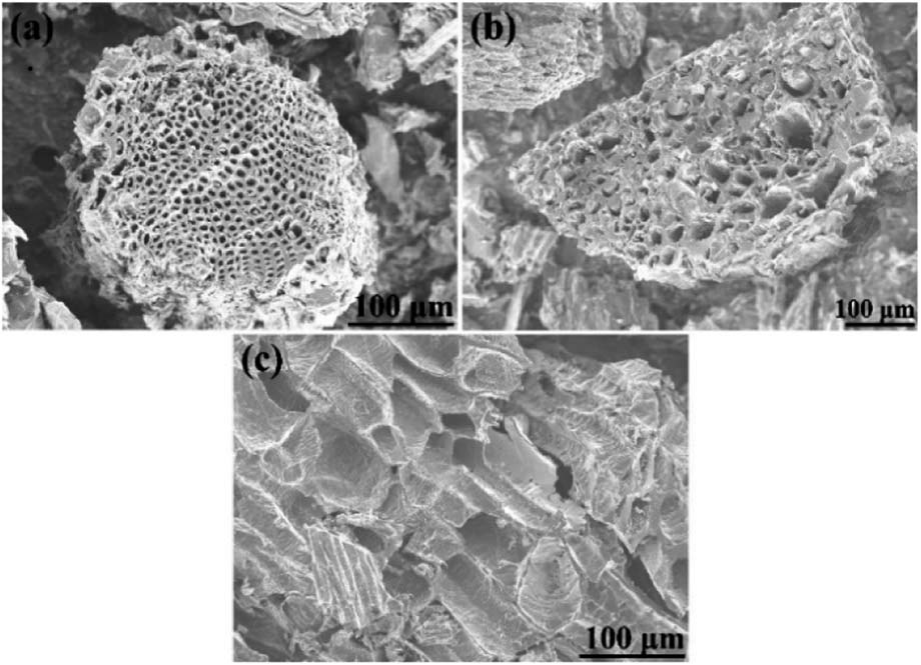
SEM images of precursor biochars of (a) – PAC, (b) – SAC, and (c) – LAC samples (before KOH activation).

Morphology of biochars produced by carbonization of cones is shown in Fig. 5. Some difference in micrometer-scale texture of samples was noted in SEM images of cone biochars. *E.g.* carbonized cones displayed a special hive-like features with multi-micrometer diameter voids inherited from precursor cones.

**Fig. 6:**
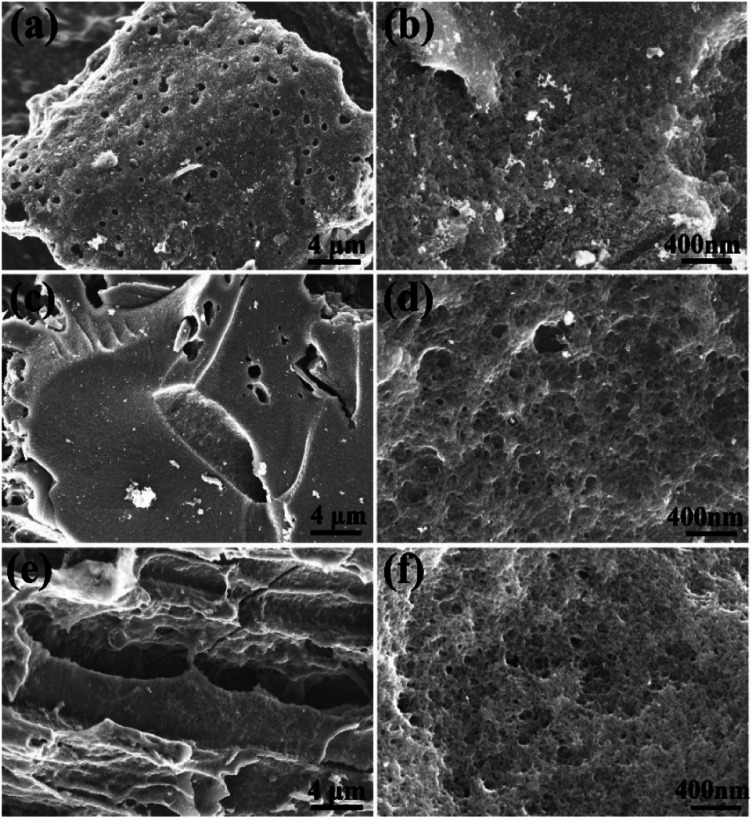
SEM images of PAC (a and b), SAC, (c and d) and LAC (e and f).

**Fig. 7:**
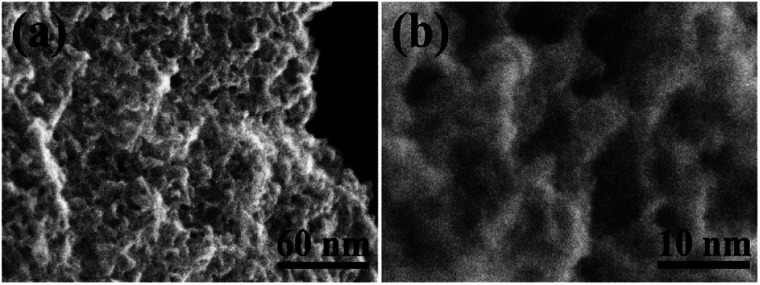
High resolution Scanning Transmission Electron Microscopy (STEM) images recorded from SAC sample: (a) lower resolution, (b) higher resolution.

However, the biochars were ground into powder before activation thus minimizing the influence of micrometer-scale architecture of these samples. High-resolution imaging provides more relevant information about the structure of materials on nm-size scale. The SEM images recorded with best resolution reveal highly porous structure with nm-size pores in all AC materials (Fig. 6) as expected from the analysis of pore size distribution provided by N_2_ isotherm (Fig. 1). The sample with highest SSA value (SAC) was also studied in more details using high resolution STEM imaging not achieved using SEM. High abundance of pores with size of few nm is revealed by these images (Fig. 7).

### Testing AC as electrode materials in supercapacitors

AC prepared for four kinds of tree cones were tested as electrode materials in supercapacitors. The tests were performed using two types of electrode forming methods. First method is straightforward compression of AC powder with binder.

**Fig. 8:**
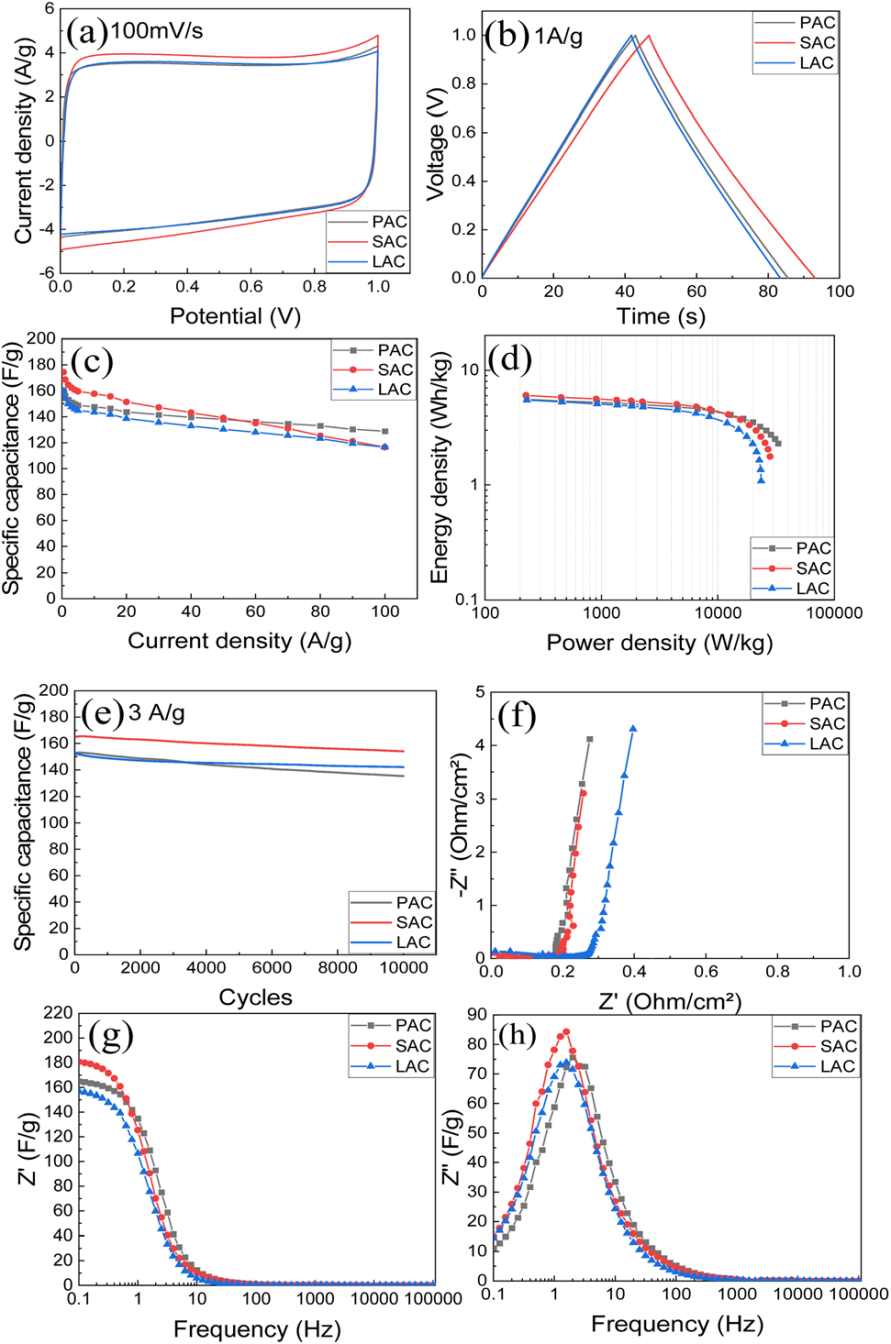
Electrochemical performance of PAC, SAC, and LAC tablets in KOH electrolyte: (a) CV at 100 mV s^−1^; (b) GCD at 1 A g^−1^; (c) specific capacitance at different current densities; (d) Ragone plots; (e) stability test for 10 000 cycles at 3 A g^−1^; (f) Nyquist plots; (g) real and (h) imaginary capacitance components.

Second method was to prepare aqueous dispersions using graphene oxide as a main surfactant and to blade coat the electrodes on current collector. Results of testing for supercapacitors prepared using pellet electrodes with 6 M KOH electrolyte are summarized in the Fig. 8 and Table 3.

**Table 3:** The electrochemical properties of activated carbons in aqueous electrolyte (6 M KOH)

Samples	Specific capacitance (F g^−1^, at 0.5 A g^−1^)	Corrected specific capacitance (F g^−1^, at 0.5 A g^−1^)	Energy density (W h kg^−1^, at 30 A g^−1^)	Power density (kW kg^−1^, at 30 A g^−1^)	Response time (s, in KOH)	Capacitance retention (%, after 10 000 cycles at 3 A g^−1^)
PAC	160.79	178.60	4.09	12.80	0.08	88.31
SAC	174.44	180.17	4.12	12.31	0.10	93.10
LAC	159.46	187.12	3.47	12.00	0.10	93.53

The CV curve (Fig. 8a) demonstrates the shape close to rectangular as expected for EDLC type of supercapacitors. Analysis of charge–discharge curves (Fig. 8b) was used to evaluate energy storage parameters of electrodes. Highest gravimetric capacitance was found for SAC sample (∼174 F g^−1^), also the sample with highest surface area and purity.

As expected, AC materials with higher amount of ash impurities showed smaller absolute values of capacitance. The AC samples showed good cycling stability with 88–93% retention after 10 000 cycles (Fig. 8e) The Nyquist plots (Fig. 8f) show low-amplitude semicircle reflecting good conductivity of electrodes and low interface resistance. The cells showed similar response in the whole measured frequency range. Impedance data were plotted also as frequency-dependent complex capacitance which allows to evaluate the efficiency of a capacitive rate response^55^ and as an imaginary capacitance plot, which peaks at a knee frequency (or its reciprocal characteristic capacitor response time *τ*) separating the domination of capacitance and resistance. The SAC electrodes show the most efficient capacitive response in agreement with highest capacitance values among other samples. The capacitive response (Fig. 8h) shows rather rapid and similar characteristic time of 0.06–0.1 s for all cone-derived electrodes. Energy storage parameters of SC with pellet AC electrodes are summarized in (Fig. 8d).

Once again, the highest energy density is observed for the AC material with highest measured surface area. Using organic electrolytes allows to achieve better energy storage parameters due to broader voltage window.

**Fig. 9:**
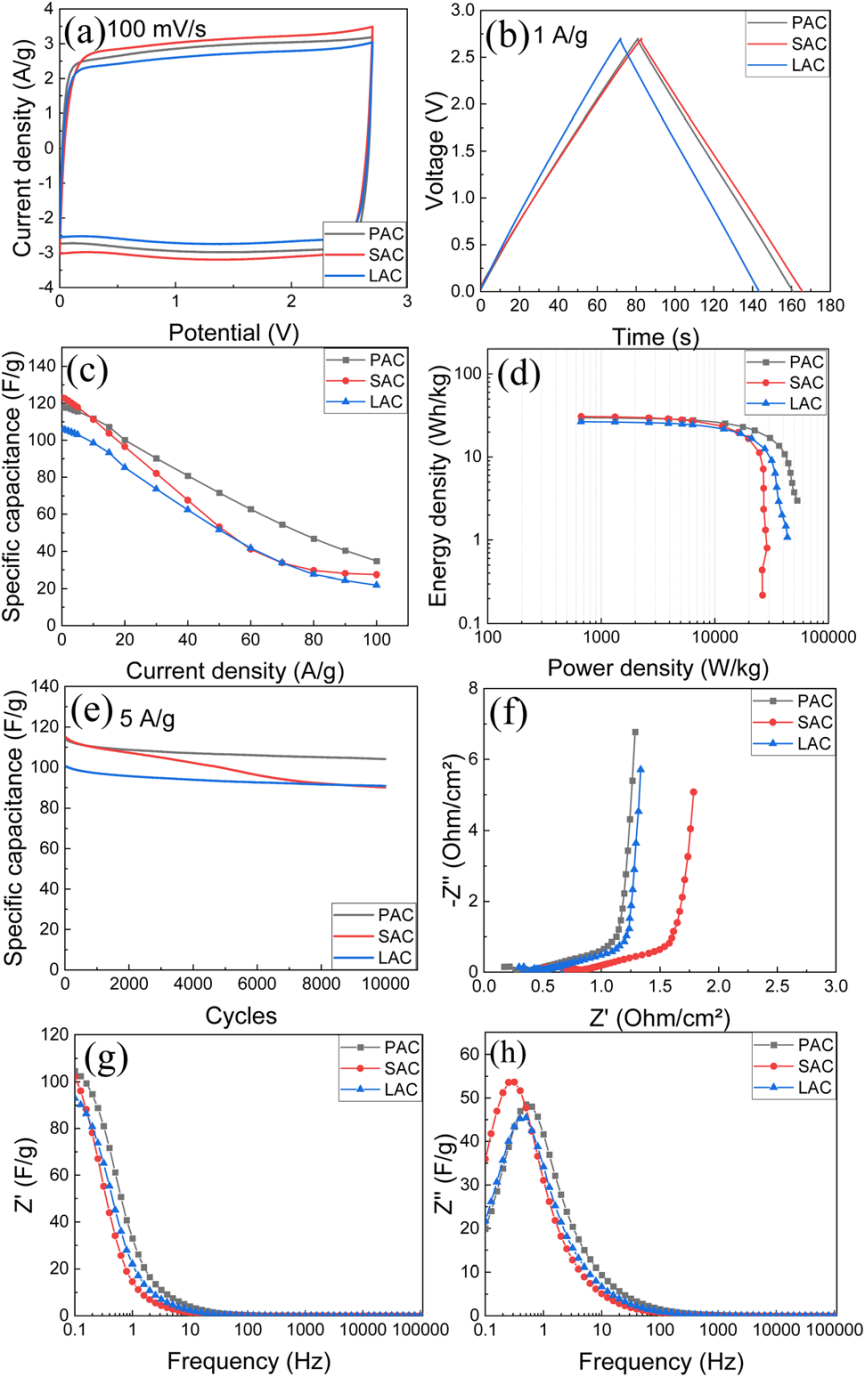
Electrochemical performance of PAC, SAC, and LAC (pellet electrodes) in organic electrolyte (1 M, TEA-BF4 in acetonitrile): (a) CV at 100 mV s^−1^; (b) GCD at 1 A g^−1^; (c) specific capacitance at different current densities; (d) Ragone plots; (e) stability test for 10 000 cycles at 5 A g^−1^; (f) Nyquist plots; (g) real and (h) imaginary capacitance components.

Therefore, we tested four samples of cone-produced AC in SC with pellet electrodes and TEA-BF_4_/acetonitrile electrolyte (Fig. 9a–h). Once again, the samples with higher SSA values generally showed better performance and higher energy storage. Excellent gravimetric capacitance of ∼122 F g^−1^ was achieved for SAC samples. Table 4 shows also values of gravimetric capacitance calculated only for carbon part of samples (excluding ash content).

**Table 4:** The electrochemical properties of activated carbons in organic electrolyte (1 M, TEA-BF4 in acetonitrile)

Samples	Specific capacitance (F g^−1^, at 1 A g^−1^)	Corrected specific capacitance (F g^−1^, at 1 A g^−1^)	Energy density (W h kg^−1^, at 30 A g^−1^)	Power density (kW kg^−1^, at 30 A g^−1^)	Response time (s, in KOH)	Capacitance retention (%, after 10 000 cycles at 5 A g^−1^)
PAC	118.01	131.08	20.93	22.43	0.08	91.28
SAC	122.53	126.55	19.82	16.13	0.50	78.19
LAC	105.61	123.93	16.71	21.11	0.10	90.10

The similarity between performances of electrodes prepared using three different bio precursors become even more obvious in this representation. Notably, the samples with the highest surface area (SAC) showed somewhat worse cycling stability and stronger drop of gravimetric capacitance at higher current density as compared to PAC sample. Ragone plot (Fig. 9d) shows that high energy and power density are achieved using cone-derived AC electrodes, with best sample showing energy density 20.9 W h kg^−1^ and power density 22.4 kW kg^−1^.

Energy storage parameters of supercapacitors prepared using blade coated water based dispersions based on AC were also analysed. Very similar electrochemical performance was observed except for somewhat smaller energy storage values as expected due to influence of added components. Fumed silica and graphene oxide are required to make the aqueous dispersions stable but these additions are not contributing to energy storage. The electrodes prepared using blade coating showed also less good cycling stability, most likely due to GO addition. Therefore, this study was mostly focused on pellet electrodes (see ESI File† for the data with performance of blade deposited electrodes).

It is interesting to note that the energy storage parameters recorded here for AC produced from essentially cost free wood waste precursors are very similar to the performance of “activated graphene” in supercapacitor devices.^3,28,29^ “Activated graphene” is produced starting from graphite oxide which is explosively exfoliated to produce rGO powder. KOH activation of rGO results is a material with BET surface area, pore size distribution and performance in supercapacitors which are very similar to those reported here for ACs. Using essentially cost free wood waste instead of expensive and still rather exotic graphite oxide as a precursor for high surface area carbons is clearly an advantage for practical applications in energy storage.

### Hydrogen sorption by cone-derived AC

Activated carbons are materials demonstrating some of the best hydrogen storage parameters as compared to other nanostructured carbons and other high surface area materials. Therefore, hydrogen sorption tests were performed with two selected samples of cone-derived AC.

Hydrogen sorption tests using volumetric method were performed for the LAC sample with SSA (2981 m^2^ g^−1^) at ambient and liquid nitrogen temperature. The same samples were tested for hydrogen storage also using Gravimetric Tank Method (GTM). Note that this sample is from different batch compared to the one described in Table 1 since relatively large samples are needed for good precision measurements in the GTM method. The excess H_2_ sorption of 0.96% (120 bar) was measured for this sample by volumetric method at ambient temperature (296 K) and 4.6 wt% at liquid nitrogen temperature. Volumetric test was also performed at ambient temperature for the sample with SSA of 2656 m^2^ g^−1^ (activated carbon from full larch cone) and showed 0.87 wt% (120 bar) excess uptake.

The volumetric method allows to measure H_2_ uptake as a surface excess value. Direct evaluation of hydrogen storage as amount of hydrogen storage inside of tank is enabled by Gravimetric Tank Method (GTM) developed in our earlier study.

**Fig. 10:**
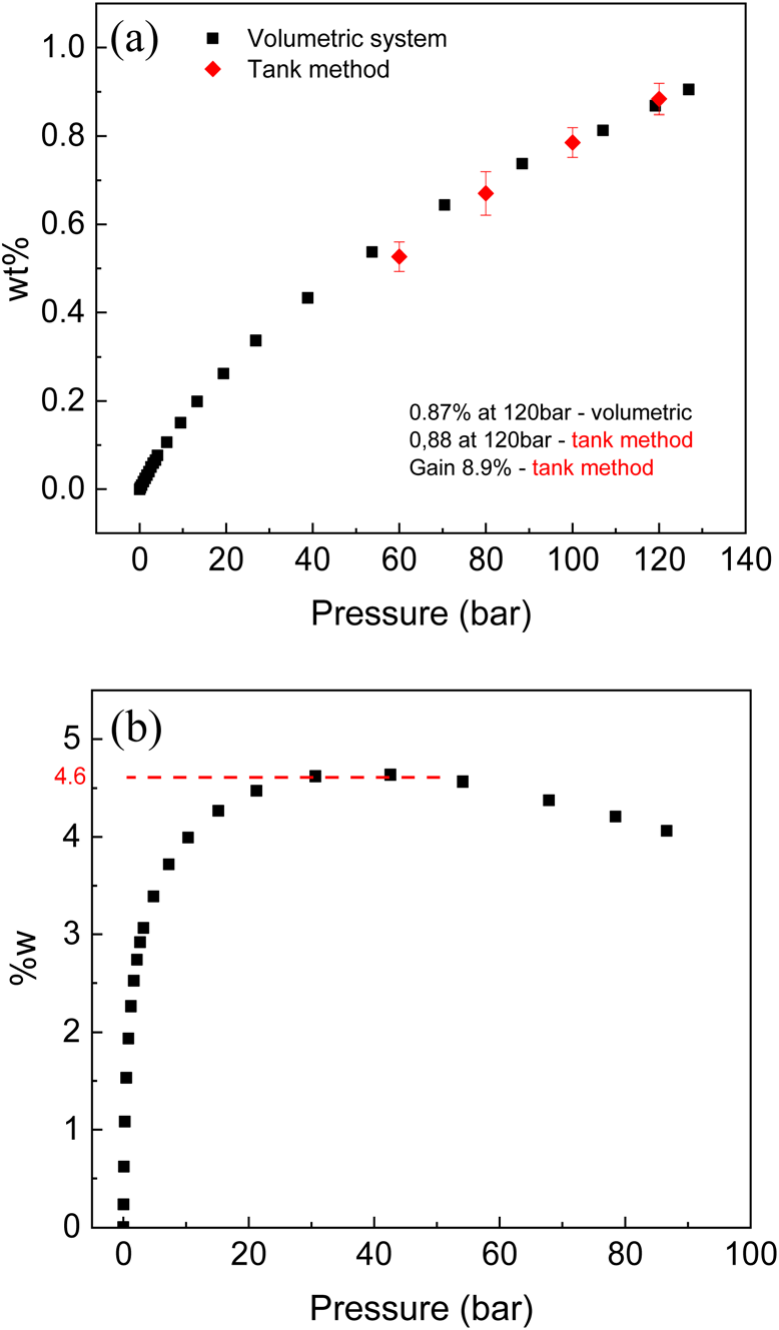
Hydrogen sorption isotherm recorded from LAC sample (SSA = 2656 m^2^ g^−1^): (a) at ambient temperature (296 K), and (b) at liquid nitrogen temperature. Red points added to the ambient temperature isotherm were obtained using gravimetric tank method.

The main goal of this method is to measure hydrogen storage Gain defined as an ability of material to increase (or decrease) amount of hydrogen stored inside of tank compared to hydrogen stored in the same volume as a pure H_2_ gas. The Gain value of 8.9% was evaluated using GTM method for LAC sample with SSA of 2981 m^2^ g^−1^. According to definition of Gain, the tank completely filled with powder of LAC at 120 bar H_2_ pressure will store 8.9% more hydrogen (by weight) compared to the same tank filled with pure gas (no material added). The GTM also allows to calculate excess wt% providing verification of isotherm recorded using volumetric method (red points in the Fig. 10a). It should be noted that high surface area AC is one of relatively few materials, which demonstrate positive values of Gain at ambient temperature. That is, adding material to the tank help to store more hydrogen due to large surface excess uptake. Many materials capable to physisorb hydrogen (*e.g.* reduced graphene oxide rGO) were demonstrated to exhibit negative gain, when adding material to the tank results in smaller total hydrogen storage.

The Gain takes into account volumetric properties of material and explicitly includes bulk density into calculation of volume occupied by material. Therefore, Gain value can be improved by compaction of powder into pellets. Low bulk density of high surface area AC (∼0.2 g ml^−1^ for sample used in this study) is typical obstacle in using this material for practical gas storage application. It is very likely that bulk density of our AC can be increased by 2–3 times using pressurizing into pellets or by using more advanced monolith compaction methods reported in literature. In this case, the Gain value is expected to increase up to 15–20% thus providing sizable increase of hydrogen storage capacity for material-filled H_2_ tanks.

**Fig. 11:**
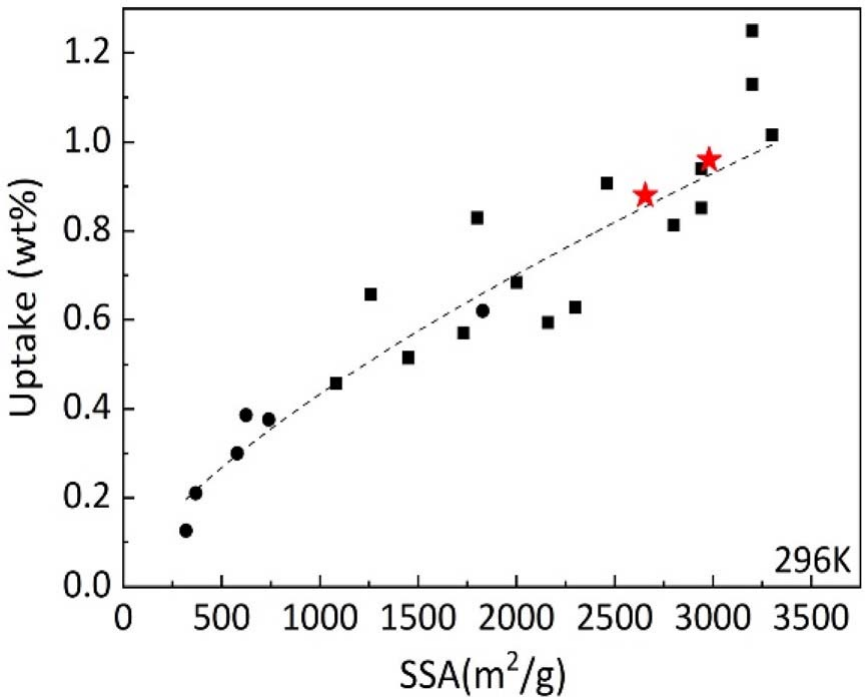
Excess hydrogen uptake measured at 120 bar H_2_ pressure and ambient temperature by various carbon materials *vs.* BET surface area. The measurements performed in this study are shown by red stars. Black symbols reflect general trend reported in our earlier study including rGO (circles) and various arGO samples produced with variations of KOH activation procedure.^10^

It is interesting to compare hydrogen uptake measured in our experiments for cone-derived AC with overall trends reported earlier for other nanostructured carbon materials. The Fig. 11 shows data points obtained earlier for hydrogen uptake at 120 bar and 296 K for several types of materials: rGO at lower SSA values, SWNT's, commercial activated carbon with SSA ∼2000 m^2^ g^−1^ and variety of “activated graphene” samples produced at different conditions. It should be noted that “activated graphene” samples with highest SSA values shown in this graph were obtained using KOH procedure (applied to rGO precursor) very similar the procedure used in this study for activation of biochars. Fig. 11 shows that hydrogen sorption properties of LAC are following general trends in H_2_ uptake *vs.* SSA values and provides values which are among the best reported. However, activated carbons are materials, which are a lot less expensive in production compared to *e.g.* CNT's or “activated graphene.” Moreover, activated carbons were produced in this study from bio precursors rather abundant in forest industry and currently considered as a waste.

## Conclusions

In conclusion, several bio precursors were used to produce AC with extremely high BET surface area (up to ∼3500 m^2^ g^−1^). It is demonstrated that identical activation procedure results in rather similar AC materials independently on selected precursor. All AC produced starting from pine, spruce, larch cones and bark/wood chips demonstrated very similar pore size distribution, high values of BET surface area and good performance as electrode materials in supercapacitors. Hydrogen sorption of AC was evaluated using volumetric method (at ambient and liquid nitrogen temperature) and gravimetric tank method (at ambient temperature). The physisorption of hydrogen is demonstrated to follow the general for all carbon materials trend *vs.* BET surface area with best sample showing 0.96 wt% excess H_2_ sorption at ambient temperature. The best AC carbon is shown to provide increase in the amount of stored hydrogen by ∼9% as compared to the tank filled with pure hydrogen gas. Our results demonstrate that details of activation procedure are more important for preparation of high surface area AC than choice of bio precursors. High quality AC can be produced starting from cost-free by-products of wood industry (*e.g.* cones, bark and wood chips) thus contributing to recycling of industrial waste.

## Author contributions

Conceptualization: A. T. Methodology: A. T., M. E. Investigation, visualization and data curation: G. L, A. I., N. B., O. A. L. Formal analysis G. L., A. T. Supervision: A. T., M. E. Writing – original draft: G. L., A. T. Writing-review and editing: all authors.

## Conflicts of interest

There are no conflicts to declare.

## Supplementary Material

RA-013-D3RA00820G-s001
