# Supplementary material for: Activated carbons with extremely high surface area produced from cones, bark and wood using the same procedure
Source: RSC Adv. 2023 May 12;13(21):14543–53. doi: 10.1039/d3ra00820g (PMC10177221; doi:10.1039/d3ra00820g)
Supplement: RA-013-D3RA00820G-s001 [file RA-013-D3RA00820G-s001.pdf]

Electronic Supplementary Material (ESI) for RSC Advances.
This journal is © The Royal Society of Chemistry 2023

Supporting information file

Activated carbons with extremely high surface area: what is more important-precursor or activation method?

Gui Li,[a] Artem Iakunkov,[a] Nicolas Boulanger,[a] Oana Andreea Lazar[b], Marius Enachescu,[b] Alejandro Grimm,[c] Alexandr Talyzin[a]

[a] Department of Physics, Umeå University, Umeå, SE-90187 Sweden
[b] Center for Surface Science and Nanotechnology University Politehnica of Bucharest Splaiul Independentei 313, Bucharest 060032, Romania
[c] Department of Forest Biomaterials and Technology, Biomass Technology Centre, Swedish University of Agricultural Sciences, Umeå SE-901 83, Sweden

1. Additional analysis of nitrogen sorption isotherms.

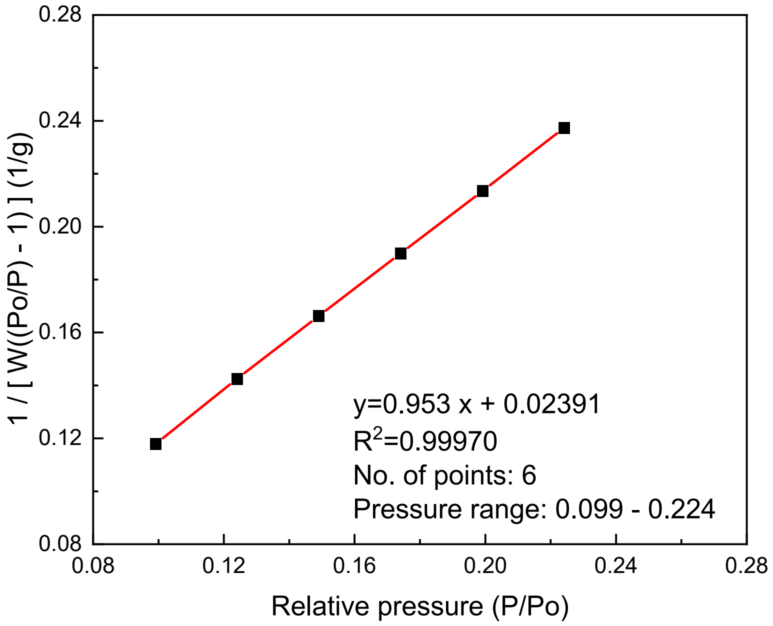

Figure S1. Example of BET plot used to estimate BET SSA of SAC sample.

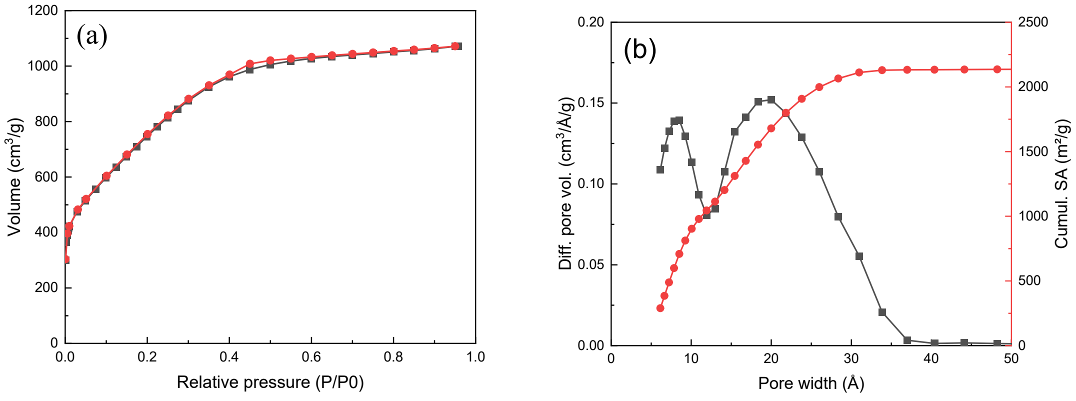

Figure S2. N_2-sorption isotherm (a) pore size distribution, (b) pore size distribution and cumulative surface area of SBAC sample (QSDFT model), BET SSA of SBAC sample is 2856 m^2/g.

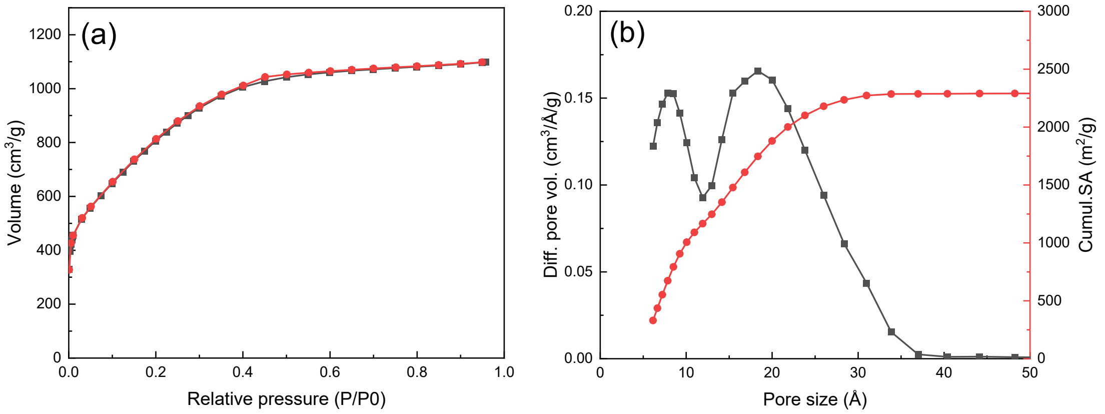

Figure S3. N_2-sorption isotherm (a) pore size distribution, (b) pore size distribution and cumulative surface area of the AC prepared using full spruce cones (3067 m^2/g).

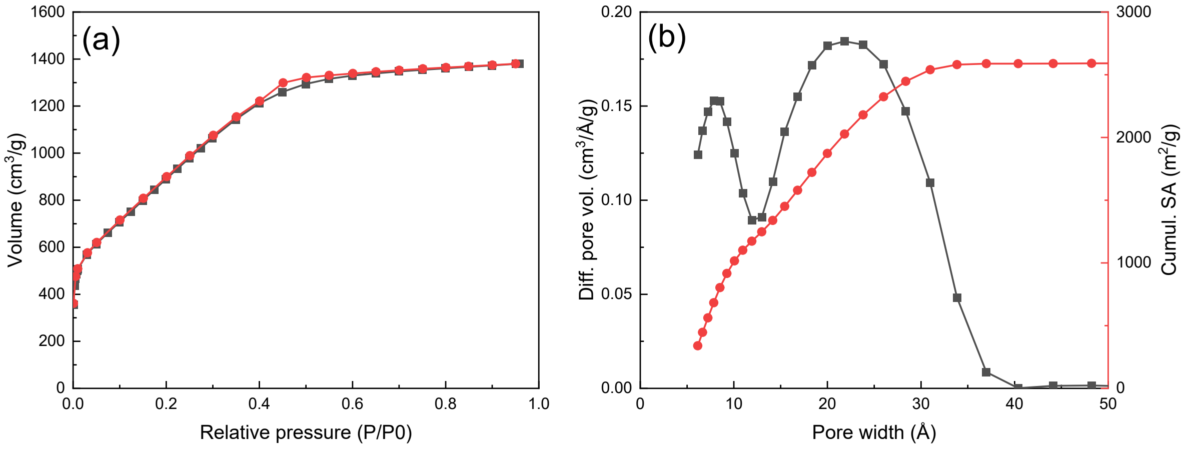

Figure S4. N_2-sorption isotherm (a) pore size distribution, (b) pore size distribution and cumulative surface area of SSAC sample (3497 m^2/g).

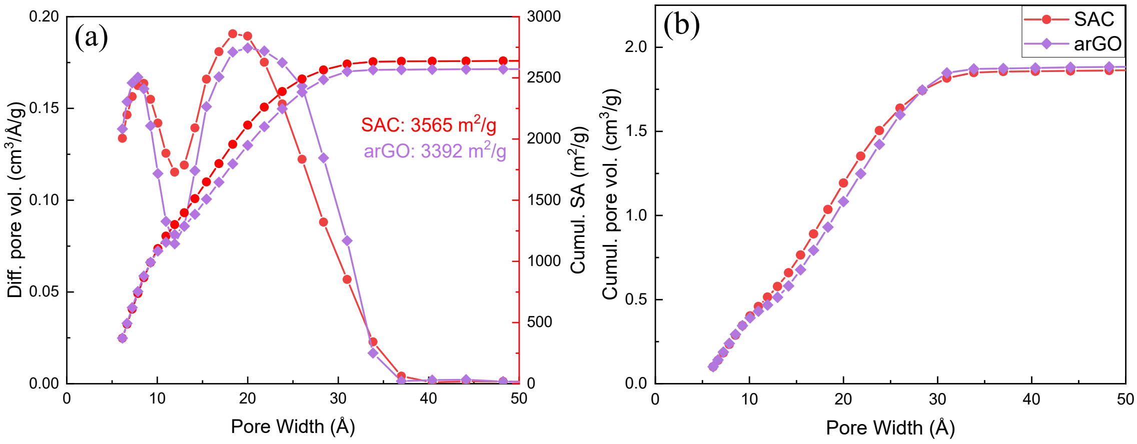

Figure S5. Analysis of N_2-sorption isotherms recorded from samples of SAC and sample of "activated reduced graphene oxide" (arGO) produced by very similar KOH activation procedure, see ref.[1] (a) pore size distribution and pore size distribution and cumulative surface area (b) Cumulative pore volume of SAC and arGO.

2. TGA data for AC materials.

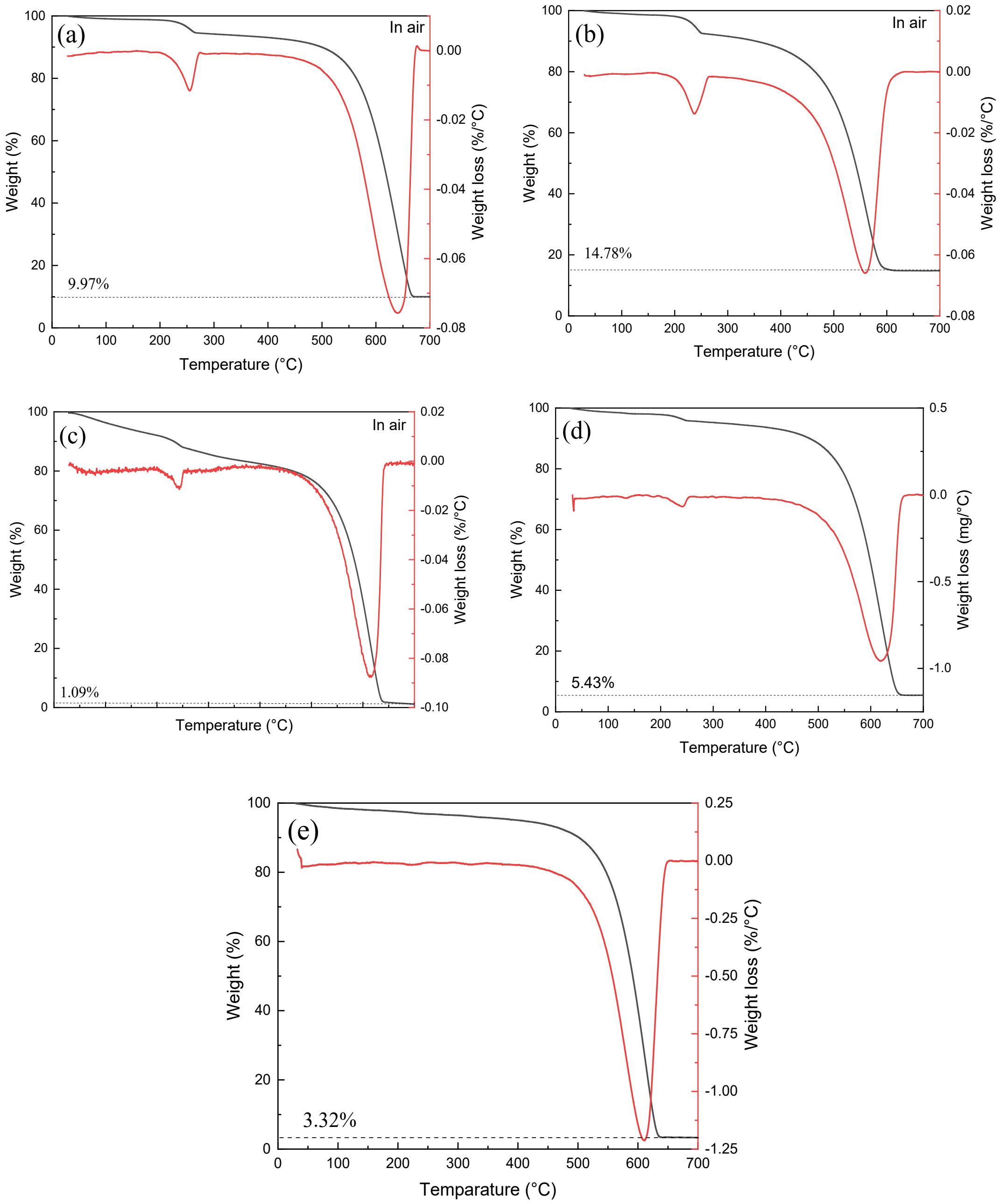

Figure S6. TGA traces recorded in air to study the thermal decomposition and ash content of samples: (a) PAC, (b) LAC, (c) SBAC; (d) full Spruce cone; (e) SSAC.

3. XRD characterization of AC materials.

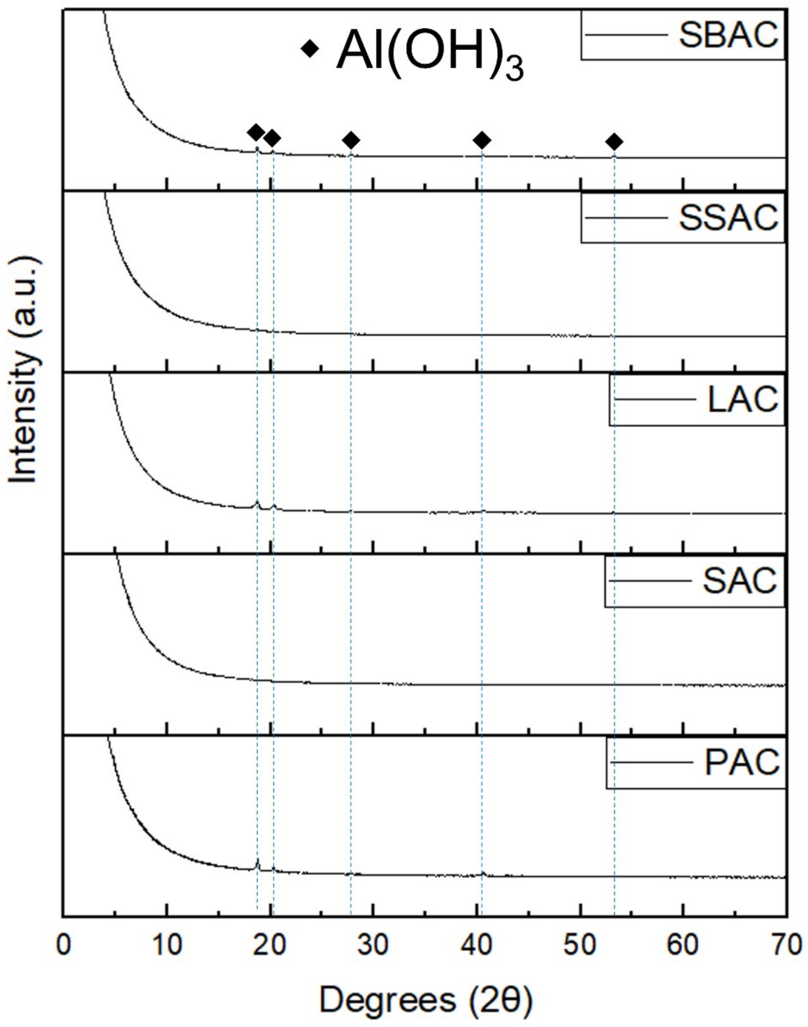

Figure S7. XRD traces recorded using CuKα radiation from samples: PAC, SAC, LAC, SSAC and SBAC. XRD reflections due to impurity of $Al(OH)_3$ are marked by diamonds.

4. Element composition of AC materials according to XPS.

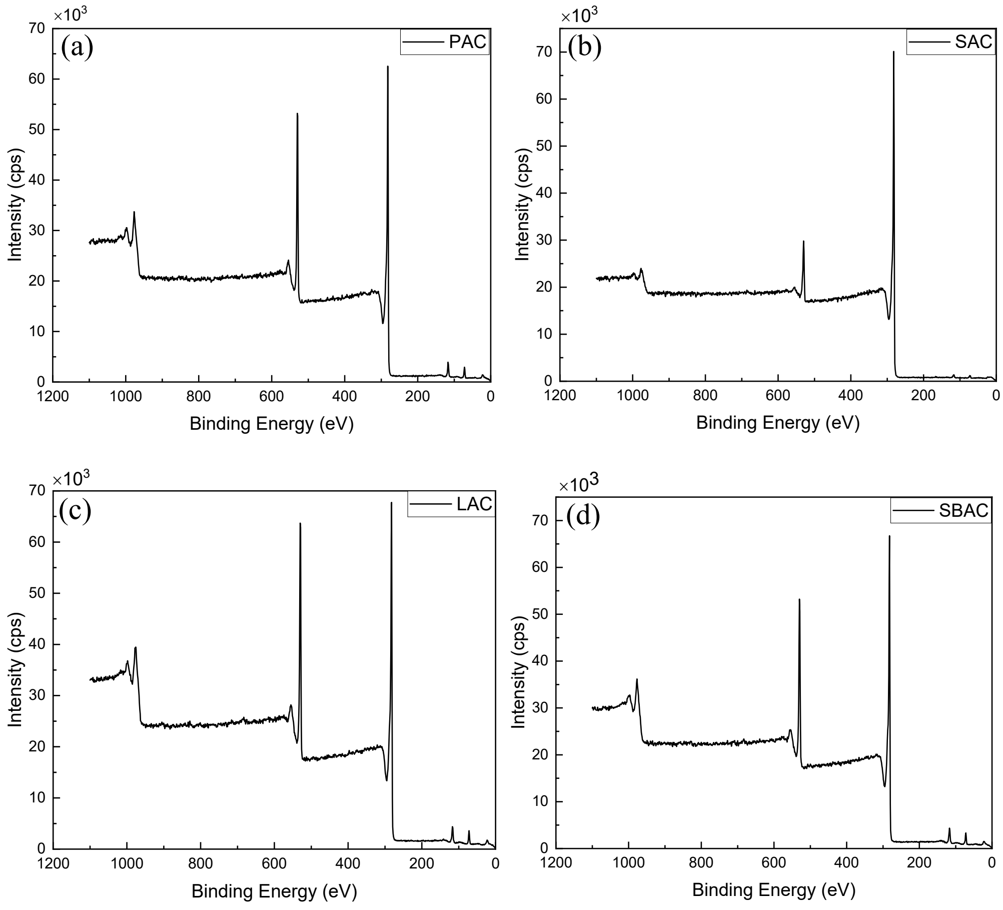

Figure S8. XPS survey scans of PAC, SAC, LAC, and SBAC.

Table 1. Element compositions of PAC, SAC, LAC and SBAC

Samples	C (at.%)	O (at.%)	Al (at.%)	$O_{\text{in C}}$ (at.%)	$C/O_{\text{in C}}$
**PAC**	67.76	14.84	4.27	2.03	33.38
**SAC**	73.40	5.89	1.10	2.60	28.7
**LAC**	64.09	16.54	4.26	3.8	17.00
**SBAC**	70.79	14.57	4.67	0.56	126.41

5. Electrochemical characterization of supercapacitors with electrodes prepared using aqueous dispersions (KOH electrolyte).

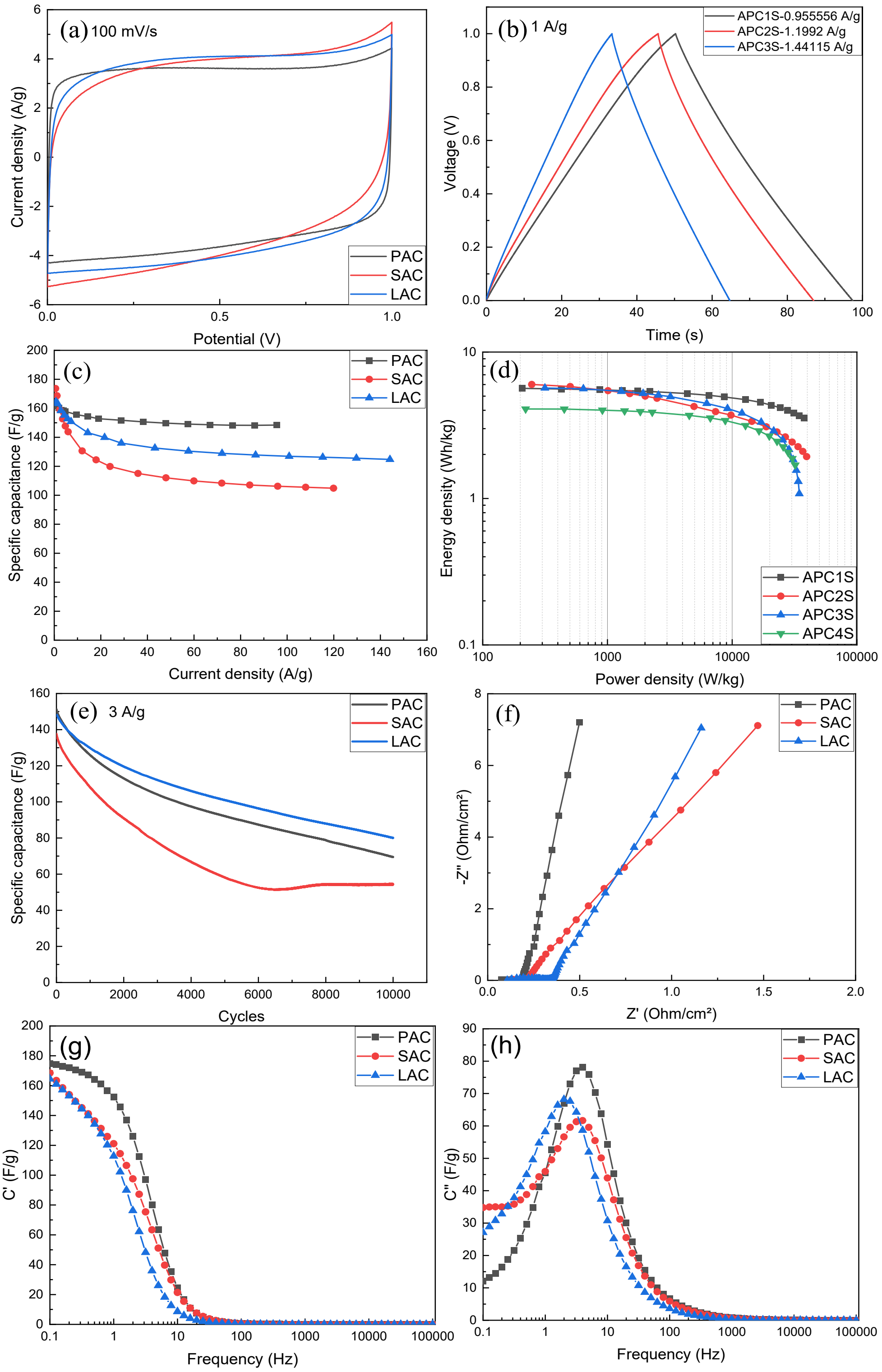

Figure S9. Electrochemical characterization of supercapacitors with PAC, SAC, and LAC electrodes prepared using blade deposition of aqueous dispersions (KOH electrolyte) : (a) CV at 100mV/s; (b) GCD at 1A/g; (c)

specific capacitance at different current densities; (d) Ragone plots; (e) stability test for 10000cycles at 3 A/g; (f) Nyquist plots; (g) real and (h) imaginary capacitance components.

6. Electrochemical characterization of supercapacitors with electrodes prepared using dispersion (organic electrolyte, TEA-BF_4)

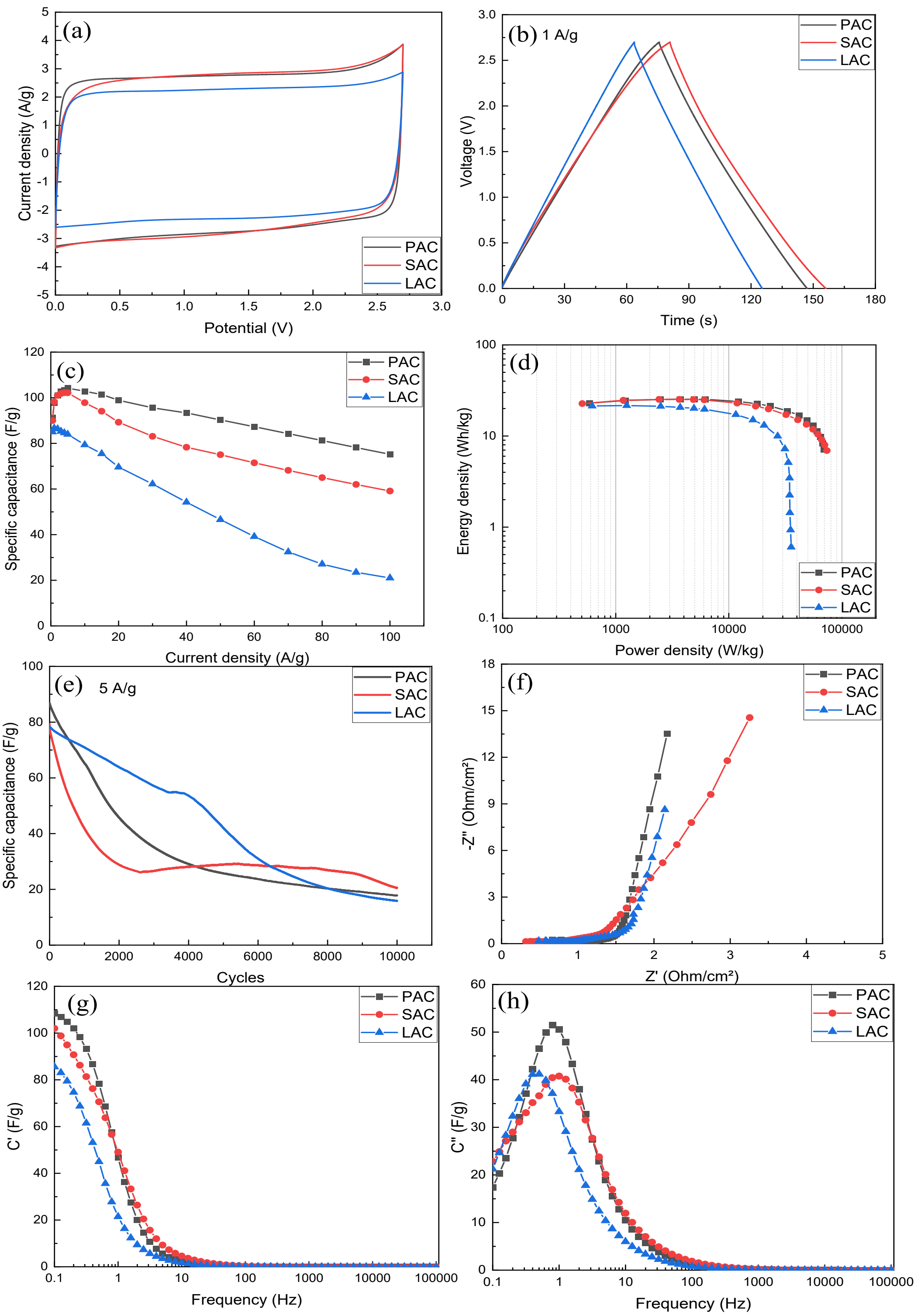

Figure S10. Electrochemical characterization of supercapacitors with PAC, SAC, and LAC electrodes prepared using blade deposition of aqueous dispersions (organic electrolyte, TEA-BF_4): (a) CV at 100mV/s; (b) GCD at 1A/g; (c) specific capacitance at different current densities; (d) Ragone plots; (e) stability test for 10000cycles at 5A/g; (f) Nyquist plots; (g) real and (h) imaginary capacitance components.

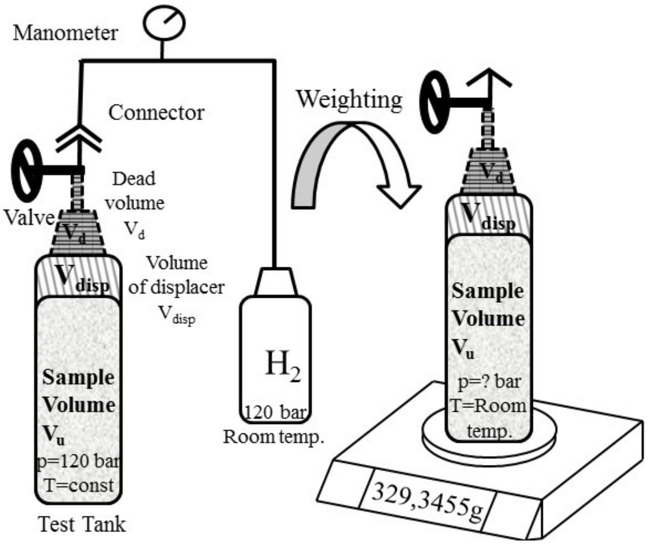

Figure S11 Scheme of Gravimetric Tank Method (GTM).[2] Small stainless steel tank can be closed with H_2 gas inside and diconnected from the source for weight measurements. The weight of hydrogen gas inside of this tank at 120 Bar is used as a reference. The weight tank is measured in empty state and after added AC sample. Then the weight is re-measured after adding hydrogen at 120 Bar. The amount of hydrogen stored in empty tank and in material-filled tank is then compared. The gain value is positive if adding material increases hydrogen storage, the gain is negative if adding material decreases amount of stored hydrogen. For example, 10% gain value means that the material –filled tank can store 10% more hydrogen by weight.

1. Klechikov, A.; Mercier, G.; Sharifi, T.; Baburin, I. A.; Seifert, G.; Talyzin, A. V., Hydrogen Storage in High Surface Area Graphene Scaffolds. *Chem Commun* **2015**, *51*, 15280-15283.
2. Iakunkov, A.; Klechikov, A.; Sun, J. H.; Steenhaut, T.; Hermans, S.; Filinchuk, Y.; Talyzin, A., Gravimetric Tank Method to Evaluate Material-Enhanced Hydrogen Storage by Physisorbing Materials. *Phys Chem Chem Phys* **2018**, *20*, 27983-27991.
